# Osmotic Demyelination Syndrome as the Initial Manifestation of a Hyperosmolar Hyperglycemic State

**DOI:** 10.1155/2014/652523

**Published:** 2014-11-09

**Authors:** Karla Victoria Rodríguez-Velver, Analy J. Soto-Garcia, María Azucena Zapata-Rivera, Juan Montes-Villarreal, Jesús Zacarías Villarreal-Pérez, René Rodríguez-Gutiérrez

**Affiliations:** ^1^Endocrinology Division, University Hospital “Dr. José E. González” and Medical School of the Autonomous University of Nuevo León, Madero and Gonzalitos s/n, 64460 Monterrey, NL, Mexico; ^2^Internal Medicine Department, University Hospital “Dr. José E. González” and Medical School of the Autonomous University of Nuevo León, Madero and Gonzalitos s/n, 64460 Monterrey, NL, Mexico

## Abstract

Osmotic demyelination syndrome (ODS) is a life-threatening demyelinating syndrome. The association of ODS with hyperosmolar hyperglycemic state (HHS) has been seldom reported. The aim of this study was to present and discuss previous cases and the pathophysiological mechanisms involved in ODS secondary to HHS. A 47-year-old man arrived to the emergency room due to generalized tonic-clonic seizures and altered mental status. The patient was lethargic and had a Glasgow coma scale of 11/15, muscle strength was 4/5 in both lower extremities, and deep tendon reflexes were diminished. Glucose was 838 mg/dL; serum sodium and venous blood gas analyses were normal. Urinary and plasma ketones were negative. Brain magnetic resonance revealed increased signal intensity on T2-weighted FLAIR images with restricted diffusion on the medulla and central pons. Supportive therapy was started and during the next 3 weeks the patient progressively regained consciousness and muscle strength and was able to feed himself. At 6-month follow-up, the patient was asymptomatic and MRI showed no residual damage. In conclusion, the association of ODS with HHS is extremely rare. The exact mechanism by which HHS produces ODS still needs to be elucidated, but we favor a rapid hypertonic insult as the most plausible mechanism.

## 1. Background

Osmotic demyelination syndrome (ODS) is a life-threatening demyelinating syndrome, which usually occurs in the setting of a rapid correction of severe chronic hyponatremia [1, 2]. Now rarely seen, ODS is a clinical syndrome characterized by altered mental status, quadriparesis, dyspnea, dysarthria, and dysphagia which all occur characteristically five to seven days after the correction of serum sodium [3]. Although the pathogenesis is not clearly understood, it is known that rapidly increasing serum osmolality shifts water out of the cells as a response to correct solute imbalance. This results in shrinkage of glial cells that can consequently lead to disruption of the blood-brain barrier allowing inflammatory mediators to enter the central nervous system damaging oligodendrocytes and myelin [4–7].

Even though ODS has been classically thought to be exclusively secondary to a rapid correction of hyponatremia, it has also been described, even though rarely, in various other situations such as malnutrition, liver transplantation, alcoholism, hypokalemia, hypophosphatemia, AIDS, lithium toxicity, hypoglycemia, and folate deficiency, among others [8–15]. In all cases a growing body of evidence demonstrates that more than sodium* per se*, the key factor, in ODS pathogenesis, is a rapid change in serum osmoles. The association of ODS with hyperosmolar hyperglycemic state (HHS) has been seldom reported with less than five cases in the literature [16–20]. Previous cases have been in the clinical scenario of concomitant hypernatremia, chronic hyperglycemia/epilepsy, and after HHS treatment. In this sense, to our knowledge, this is the first case of ODS as the opening syndrome of HHS.

Herein we present the case of a patient with HHS who presented with ODS as the initial manifestation of his illness. A review of previous cases and pathophysiological mechanisms involved in ODS secondary to HHS will be presented and discussed.

## 2. Case Presentation

A 47-year-old man arrived to the emergency room due to generalized tonic-clonic seizures and altered mental status. He had a history of long-standing uncontrolled type 2 diabetes treated irregularly with insulin and metformin (HbA1c 10.1%), with no known microvascular or macrovascular complications. He had no history of alcohol consumption and twenty-four hours earlier he referred nocturia, polyuria and polydipsia. On the day of admission, while asleep, he suddenly developed three consecutive episodes of generalized tonic-clonic seizures along with urinary sphincter loss and subsequent postictal state.

On physical examination he was hemodynamically stable with a blood pressure of 130/80 mmHg, heart rate of 85 beats per minute, respiratory rate of 20 per minute, temperature 36.5°C, and room-air oxygen saturation of 98%. His body mass index was 32.2. Mucous membranes were remarkably dry. The patient was lethargic and had a Glasgow coma scale of 11/15, muscle strength was 4/5 in both lower extremities, and deep tendon reflexes were diminished. Cranial nerves were normal, sensitivity was preserved, and meningeal signs and primitive reflexes were absent. Due to the patient status cerebellum function could not be evaluated. Finger-stick glucose could not be recorded due to high blood glucose levels and venous blood gas analysis reported pH 7.36,* P*CO_2_ 42 mmHg,* P*O_2_ 32 mmHg, lactate 1.2 mmol/L, and bicarbonate 23.7 mEq/L. Urinary and plasma ketones were negative. Hydration with intravenous normal saline solution at 500 mL/h and an insulin infusion at 0.14 U/kg were started. Plasma glucose was 838 mg/dL (46.5 mmol/L), blood nitrogen urea 21 mg/dL, creatinine 1.1 mg/dL with a MDRD calculated glomerular filtration rate of 71 mL/min, serum sodium 133 mmol/L, and chlorine 89 mmol/L. Calcium, potassium, phosphorous, magnesium, hemoglobin, white blood count, and platelets were all within normal limits. Serum albumin was 2.1 g/dL, alkaline phosphatase 192 UI/L, alanine aminotransferase 19 U/L, aspartate aminotransferase 21 U/L, total bilirubin 0.8 mg/dL, and serum osmolality 320 mOsm/kg (Table 1).

**Figure 1:**
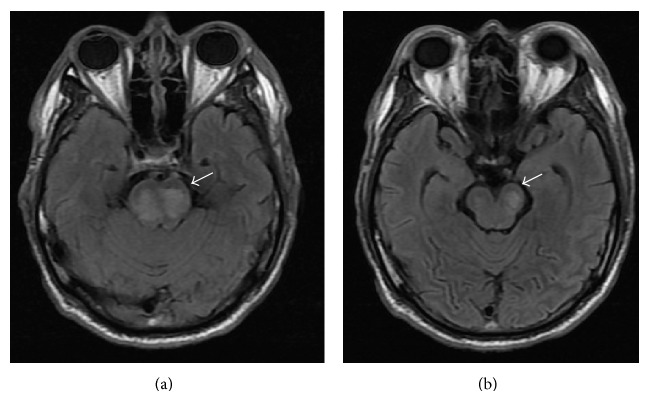
MRI with increased signal intensity on T2-weighted FLAIR images in the medulla oblongata and central pons (arrows).

Head computed tomography, electrocardiogram, and chest X-ray were normal. The patient after 24 hours of treatment continued with altered mental status and paraparesis progressed (3/5). Plasma glucose was lowered down carefully at a rate of 30–40 mg/dL/hr and was within 200–300 mg/dL [21]. Serum osmolality dropped down to 305 mOsm/Kg (Table 1). Twenty-four hours after admission a brain magnetic resonance imaging (MRI) was ordered and revealed increased signal intensity on T2-weighted FLAIR images with restricted diffusion in the medulla and central pons (Figure 1). Mammillary bodies, thalamus, third ventricle, and both hemispheres were respected. Lumbar puncture was normal, electroencephalogram showed a nonspecific wave activity, and urinary and serum toxicology panel were negative. During all his admission serum sodium was documented to be within normal range. ODS was diagnosed and aggressive supportive therapy was started. During the next 3 weeks the patient progressively regained consciousness and muscle strength and was able to feed himself. At 6-month follow-up, the patient was asymptomatic and MRI showed no residual damage.

## 3. Discussion

This case illustrates, to our knowledge, the first reported case of ODS as the initial manifestation of HHS. Osmotic demyelination was first described by Adams et al. in 1959 in a series of four cases that presented paresis, pseudobulbar paralysis, and the distinctive myelin loss in the pons, attributed to alcoholism or malnutrition [22]. It was not until the mid-1970s that routine electrolyte tests started to be measured, that the link between chronic hyponatremia and its rapid correction was made. Since then it is not infrequent that many clinicians associate rapid correction of hyponatremia as the sole cause of ODS. Nevertheless, it is now well known that a variety of other medical conditions (where an osmotic shift has not been identified) such as alcoholism, malnutrition, cirrhosis, liver transplantation, hypokalemia, hypophosphatemia, and hypomagnesaemia, AIDS, folate deficiency, psychogenic polydipsia, beer potomania, refeeding syndrome, dialysis disequilibrium syndrome, hyperemesis gravidarum, sepsis, malignancy, lithium toxicity, prolonged diuretic use, and hypoglycemia have also been associated with ODS [6–12].

The association of ODS secondary to HHS has been seldom reported. See Table 2. In 1989, McComb et al. reported the first case of a ODS related to a HHS. Since admission, the patient presented with hypernatremia (169 mEq/L) and despite prompt and aggressive treatment serum sodium continued increasing (188 mEq/L). After three weeks the patient died and diagnosis of ODS was based on autopsy findings [16]. In 2008, O'Malley et al. reported a case of a 49-year-old woman with no previous history of type 2 diabetes that presented with glucose of 1910 mg/dL. The patient had a rapid drop of over 900 mg/dL in less than 6 hours and serum sodium at that same time went from 134 mmol/L to 159 mmol/L. Later on, the patient developed pneumonia, sepsis, and multiorgan failure and 9 days after admission presented a flaccid quadriparesis, pseudobulbar palsy, dysarthria, and impaired swallowing. An MRI confirmed the diagnosis and the patient was discharged from hospital 90 days after admission with almost complete recovery [17]. Later on Burns et al. described the case of a 93-year-old man who had an initial glucose of 524 mg/dL and a serum osmolality of 317 mOsm/kg. Neurological examination and serum sodium were normal. Hyperglycemia and osmolality were corrected within 24 hours and 2 days after presentation he developed marked gait ataxia and mild dysarthria. MRI confirmed the diagnosis of ODS and at 1-month follow-up showed marked improvement in his gait unsteadiness [18]. More recently, Mao et al. reported the case of a 55-year-old man who had a history of multiple focal seizures 3 weeks before hospitalization. On admission he presented with focal continuous seizures, fever, right-sided hemiplegia, and absent tendon reflexes. Glucose was 685 mg/dL and serum osmolality was 318 mOsm/L. Serum sodium was within normal range and on 8 hours after HHS treatment the seizures ceased. Two days later the patient regained consciousness, muscle strength improved, and he was discharged without any neurological manifestations [19]. Finally, in 2013, Guerrero et al. described the case of a 23-year-old man that developed ODS days after the treatment of HHS. Surprisingly, no sodium values, serum osmolality, or outcome was mentioned in the manuscript [20]. As described above, in four of the previous cases ODS occurred after the treatment and correction of HHS and, in the other, the patient had almost a month with seizures before developing HHS. In our case, characteristic symptoms related to ODS, confirmed by typical MRI images, were the initial manifestation of an HHS that likely developed acutely. Alterative diagnoses were ruled out, serum sodium levels were documented to be within normal range during all hospitalization, and, after the HHS resolved and supportive therapy was initiated, neurological symptoms progressively disappeared.

Although the pathogenesis of ODS is not clearly understood it is known that rapidly increasing serum osmolality shifts water out of the cells as a response to correct solute imbalance. This serves as a protective mechanism from swelling during chronic conditions of hypoosmolality that usually takes two days to be completed. In the absence of hyponatremia ODS is proposed to occur as a result of a relatively hypertonic insult in which ODS can result if the serum or the extracellular space becomes hypertonic faster than the rate at which the brain cells can compensate [3–5, 23, 24]. This consequently leads to disruption of the blood-brain barrier allowing inflammatory mediators to enter the central nervous system and damage oligodendrocytes, which may further release myelin toxin and produce vasogenic edema in the central pons [25]. Nevertheless, the breakdown concept of the blood-brain barrier as a result of osmotic stress has also been subject to debate. It has been postulated that osmotic injury may result in the release of nitric oxide or other agents harmful to tight junctions. In addition to osmotic injury and edema, the oligodendrocytes may be damaged by toxins released by injury of endothelial cells [26]. In our case we favor the hypothesis that the hypertonic insult was the cause of ODS. It is likely that the HHS developed fast enough (during the previous day to admission) that oligodendrocytes were not able to adapt. The possibility that ODS was secondary to our treatment is plausible and cannot be excluded. However, it seems unlikely because the patient since his arrival had clinical characteristics that were compatible with ODS. Furthermore the treatment plans resulted in improvement and ultimately in full-recovery.

The clinical manifestations of ODS may vary considerably depending on the degree of pontine involvement and the presence of extrapontine lesions; paraparesis, quadriparesis, dystonia, dysphagia, ataxia, tremor, catatonia, encephalopathy, locked-in syndrome, delirium, seizures, and coma have been described. Neuropsychological findings such as attention, memory, and decision-making disturbance and even psychotic symptoms are rare but have been associated with ODS. These clinical manifestations usually occur five to seven days after the hypertonic insult but had also been described to appear after two or more weeks [27]. It is important to remember that even though diagnoses of ODS can be made in the setting of rapid hyponatremia correction or a hypertonic insult in conjunction with characteristic clinical and radiological findings, alternate diagnoses have to be ruled out. The differential diagnosis for ODS would include stroke, primary brain tumors, metastases, encephalitis, meningitis, radiotherapy, chemotherapy, Wernicke encephalopathy, hepatic encephalopathy, and other demyelinating conditions such as multiple sclerosis [24]. In our case, all of these conditions were excluded.

There is no specific treatment for nonsodium dependent ODS rather than to treat the underlying illness and to initiate supportive measures. Nevertheless, it is important to follow the same concept, as with sodium, that whatever the hypertonic insult is it must be lowered carefully and gradually [1, 3, 28]. Treatment with steroid bolus, intravenous immunoglobulin, intravenous thyrotropin-releasing hormone, or plasma exchange had also been used in some ODS cases but additional studies are warranted before their implementation in clinical practice [28–31]. Although ODS has been previously thought as a devastating condition, recent literature shows that over 50% of the patients recover either completely or with minimal disability [32]. Thus, prompt diagnosis and appropriate supportive measures are mandatory.

## 4. Conclusion

In conclusion, the association of ODS with HHS has been seldom reported. We have described the case of a ODS with the characteristic clinical and radiological findings as the initial manifestation of HHS. The exact mechanism by which HHS produces ODS still needs to be elucidated but we favor a rapid hypertonic insult as the most plausible mechanism.

## Figures and Tables

**Table 1:** Laboratory measures during hospitalization.

Day	1	2	3	4	5	6	7	Normal range
Glucose	838	510	210	90	256	226	152	70–100 mg/dL
Sodium	133	138	136	138	132	132	134	135–145 mmol/L
Potassium	4.9	3.7	3.7	3.8	4.3	3.9	4.6	3.5–5 mmol/L
Chlorine	89	103	106	107	106	108	109	101–111 mmol/L
BUN	21	28	33	23	22	23	24	7–20 mg/dL
Creatinine	1.1	1.1	1.6	1.2	1.4	1.3	1.4	0.6–1.4 mg/dL
Osmolality	320	314	295	289	286	285	285	275–290 mOsm/kg
Anion gap	21	20	15	10	12	12	13	8–122 mEq/L

BUN denotes blood nitrogen urea.

**Table 2:** Reported cases of CMP associated with HHS.

Author^*^	Age yr.	Gender	Glu	Osm	Ketones	Na^∧^	Na^ç^	MRI	TASA	Outcome
McComb et al. [16]	54	F	954	NS	Negative	169	188	No	NM	Died
O'Malley et al. [17]	49	F	1910	399	Trace	134	166	Yes	9 days	ACFR
Burns et al. [18]	93	M	524	343	Negative	137	140	Yes	2 days	ACFR
Mao et al. [19]	55	M	685	318	Negative	134	NS	Yes	0 days^+^	NED
Guerrero et al. [20]	25	M	>700	NS	NS	NS	NS	Yes	NS	NS
This report	47	M	838	320	Negative	133	138	Yes	0 days	NED

^*^Reference.

^+^Patient had history of multiple focal seizures 3 weeks before admission.

CPM, central pontine myelinolysis; HHS, hyperosmolar hyperglycemic state; Glu, glucose on admission; Osm, osmolality; Na^∧^, sodium at admission; Na^ç^, maximum sodium; MRI, magnetic resonance imaging; TASA, time after admission of symptoms; NM, not mentioned; ACFR, almost complete functional recovery; NED, no evidence of disease.

Glu (mg/dL); Osm (mOsm/kg); Na (mEg/L).

## Abbreviations

ODS: Osmotic demyelination syndrome
HHS: Hyperosmolar hyperglycemic state
MRI: Magnetic resonance images.
